# Experimental Study on the Thermal Start-Up Performance of the Graphene/Water Nanofluid-Enhanced Solar Gravity Heat Pipe

**DOI:** 10.3390/nano8020072

**Published:** 2018-01-28

**Authors:** Shanguo Zhao, Guoying Xu, Ning Wang, Xiaosong Zhang

**Affiliations:** Key Laboratory of Energy Thermal Conversion and Control of Ministry of Education, School of Energy & Environment, Southeast University, Nanjing 210096, China; shanguozhao@outlook.com (S.Z.); 220140398@seu.edu.cn (N.W.); rachpe@seu.edu.cn (X.Z.)

**Keywords:** nanofluid, heat pipe, start-up characteristics, solar collection

## Abstract

The solar gravity heat pipe has been widely used for solar thermal water heating because of its high efficient heat transfer and thermal diode characteristics. Operated on fluctuant and low intensity solar radiation conditions, a solar gravity heat pipe may frequently start up. This severely affects its solar collection performance. To enhance the thermal performance of the solar gravity heat pipe, this study proposes using graphene/water nanofluid as the working fluid instead of deionized water. The stability of the prepared graphene/water nanofluid added with PVP was firstly investigated to obtain the optimum mass ratios of the added dispersant. Thermophysical properties—including the thermal conductivity and viscosity—of nanofluid with various graphene nanoplatelets (GNPs) concentrations were measured at different temperatures for further analysis. Furthermore, based on the operational evaluation on a single heat pipe’s start-up process, the performance of nanofluid-enhanced solar gravity heat pipes using different concentrations of GNPs were compared by using water heating experiments. Results indicated that the use of 0.05 wt % graphene/water nanofluid instead of water could achieve a 15.1% and 10.7% reduction in start-up time under 30 and 60 W input heating conditions, respectively. Consequently, a higher thermal efficiency for solar collection could be expected.

## 1. Introduction

Heat pipe solar collectors, which use the heat pipe as a core component of heat transfer, have been widely used in solar thermal water heating applications. The structure types of heat pipe solar collectors were mainly divided into (1) evacuated-tube heat pipe and (2) flat-plate heat pipe solar collectors. In both types of solar collectors, the evaporation sections of heat pipe arrays are placed inside the vacuum tubes or on the flat-plate solar absorber, transfers the absorbed solar heat to the condensing section. A gravity heat pipe (also called two-phase closed thermosyphon) transferring heat only in one direction acts as a thermal diode to avoid reverse heat transfer loss. Deionized water, commonly used as the working fluid, has a liquid-steam phase transition inside the sealed heat pipe. Therefore, a gravity heat pipe solar collector could achieve higher thermal efficiency and allow more pressure-bearing operations [1,2] compared with a conventional solar collector. 

As an enhanced heat transfer technology, the addition of nanoparticles to a base fluid could significantly improve the fluid’s thermal conductivity, meanwhile cause a flow feature change and enhance the convective, boiling and condensation heat transfer coefficients [3,4,5,6,7,8]. With regards to the nanofluid-enhanced heat pipe heat transfer, most previous studies have confirmed that the thermal performances of different types of heat pipes could be improved by using various nanofluid as the working fluid [9,10,11,12,13]. For example, Menlik T. et al. [11] experimentally demonstrated that the performance of a heat pipe was improved by 26% under 200 W heating power when MgO/water nanofluid was charged into the system instead of water. Wang Z.P. et al. [12] experimentally investigated the thermal characteristics of a miniature loop heat pipe applied for the cooling of high power density electronics. They concluded that a reduction of 21.7% in the total thermal resistance and an increase of 19.5% in the evaporative heat transfer coefficient were achieved, respectively, when using the copper/water nanofluid of 1.0 wt % concentration. The study carried out by Mehrali M. et al. [13] indicated that a maximum thermal resistance reduction of 58.6% were observed for grooved copper heat pipes using nitrogen-doped graphene nanofluid with a concentration of 0.06 wt %. Apart from experimental research, entropy generation analysis method was used to evaluate the effect of using nanofluid on the heat pipes’ performances. Tharayil T. et al. [14] performed the thermodynamic analysis and experimental evaluation on a miniature loop heat pipe. It is found that the use of nanofluid diminished the entropy generation and increased the second law efficiency and the dominant factor causing the entropy generation was heat transfer. While, Singh et al. [15] suggested that the entropy generation due to friction is dominant in small diameter tubes. Therefore, guidance for the further structure optimization from the thermodynamic point of view was provided [16]. 

However, it is not always advantageous in every working condition; adding nanoparticles may also lead to deterioration of the heat pipe’s thermal performances [17]. Nucleate boiling heat transfer of alumina nanofluid was found be increased for the smooth surface but unchanged or even decreased for the rough surface. It is because that deposited nanoparticles may reduce the roughness and nucleation site density of the heated surface [18,19,20]. 

Differs from a loop heat pipe, a gravity heat pipe does not have separate liquid and vapor flow line. What’s more, the gravity heat pipe employed in a solar collector has a long evaporation section placed in a vacuum tube or flat-plate solar absorber and a stubby condensing section for heat release. Therefore, the operating characteristic of the heat pipe solar collector differs from the common heat pipe heat exchanger. Many works of literature have reported the applications of nanofluid in solar thermal collectors [21,22,23,24,25,26]. Lu et al. [26] investigated the thermal performance of an open thermosyphon using nanofluid for high-temperature evacuated tubular solar collectors. Generally, most of the related research focused on the heat transfer enhancement that occurs when nanofluid is used in heat pipes. 

Due to the large fluctuation and relatively low heat flux in solar radiation, the heat pipe’s start-up characteristics under a low and unstable heat flux condition has a significant effect on the overall performance of the heat pipe solar collector. However, research on the start-up characteristics of solar heat pipes and, consequently, their effect on solar collection efficiency is still relatively little thus far.

In this paper, a kind of solar gravity heat pipe where graphene/water nanofluid is used as the working fluid was studied. Firstly, the thermal properties of different concentrations of the prepared graphene/water nanofluid were measured. This was followed by an experimental investigation on the thermal start-up characteristics of a single solar gravity heat pipe under different working conditions. Finally, the thermal performances of the nanofluid-enhanced solar gravity heat pipes using different concentrations of graphene nanoplatelets were comparatively analyzed with that of a conventional heat pipe which uses deionized water as the working fluid.

## 2. Preparation and Properties of Graphene/Water Nanofluid

### 2.1. Preparation of Graphene/Water Nanofluid

Graphene is a crystalline allotrope of carbon whose carbon atoms are densely packed in a regular atomic-scale hexagonal pattern. It has attracted widespread attention because of its excellent thermal conductivity and potential for thermal management applications. The thermal conductivity of graphene nanoplatelets (GNPs) is reported to be as high as 1500 to 5000 W/(m·K) [27], whose value is determined by the graphene quality and processing conditions. Also, the mixture of GNPs and distilled water displays a significant thermal conductivity enhancement compared to the base fluid [28,29]. Further, due to the two-dimensional structure, its heat transfer properties are expected to be different from the exiting nanoparticles and one-dimensional carbon nanotubes. Therefore, stable homogeneous grapheme/water nanofluid is prepared by dispersing GNPs in deionized water and used as the working fluid of gravity heat pipes in the present work. 

**Figure 1:**
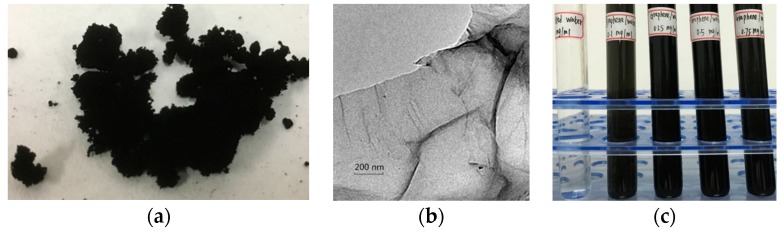
Photographs of graphene power and prepared graphene/water nanofluid. (**a**) Graphene powder used in the experiment. (**b**) SEM image of a graphene nanoplatelets (GNPs). (**c**) Prepared graphene/water nanofluid with different GNPs concentrations.

The GNPs has a particle size of 0.5–2 μm, a thickness of 0.8–1.2 nm, a single layer rate of about 80% and surface area of 800–900 m^2^/g measured using a chemical analysis method. Figure 1a is a photograph of the graphene powder. A transmission electron microscope (TEM) (FEI Company, Hillsboro, OR, USA) micrograph of the GNPs is shown in Figure 1b. It is evident that the graphene nanoplatelet has a complete single laminated structure. The base fluid of the nanofluid prepared in this paper was deionized water. Previous research has indicated that polyvinylpyrrolidone (PVP) has a strong affinity with graphite surfaces and better dispersibility [30]. Therefore, PVP was chosen as the dispersant in the present work. 

Using a two-step method, GNPs were slowly added to the deionized water through the process of magnetic stirring. Then, PVP was added to the graphene/water suspension under ultrasonic oscillation conditions. Figure 1c shows the prepared graphene/water nanofluid mixtures with different GNPs concentrations.

### 2.2. Dispersion Stability

To investigate the effect of the addition of PVP on the dispersion stability of the graphene/water nanofluid, the spectrum absorbance of nanofluid with different PVP to GNPs mass ratios were measured using an ultraviolet-visible spectrophotometer. PVP to GNPs with seven different mass ratios (1:1, 2:1, 3:1, 4:1, 5:1, 6:1 and 7:1) were added to the same volume of deionized water. Because of the strong extinction effect, graphene/water nanofluid was diluted with deionized water before absorbance measurements were taken. 

There is a linear relationship between the absorbance at a specific wavelength and the concentration of nanoparticles within the suspension fluid, which is represented in Equation (1) [31]. According to Equation (1), the higher absorbance is, the less agglomeration and sedimentation occurs within a given standing time. With higher absorbance, there is also better dispersion and stability.

(1) $$A=\mathrm{lg}\left(I_{0}/I\right)=\varepsilon bc$$

where *A* is the absorbance in Abs; *I*_0_ and *I* is the intensity of the incident and transmitted light, respectively, in W/m^2^; *ε* is the molar absorptivity in L·mol^−1^·cm^−1^; *b* is the optical distance in centimeters; and *c* is solute molarity in mol·L^−1^.

**Figure 2:**
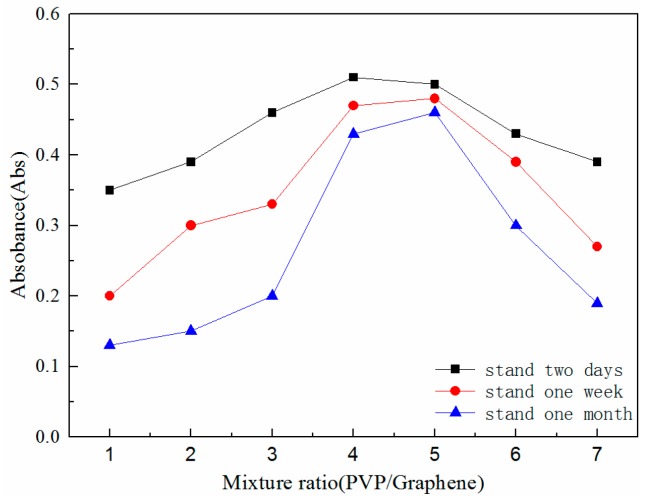
Absorbance of graphene/water nanofluid.

Figure 2 shows the measured absorbance of nanofluid with different mass ratios of PVP to GNPs after standing for different amounts of time (i.e., two days, one week, or a month). The specific wavelength for measurement in this study was 400 nm. It is evident that when the mass ratio of PVP to GNPs was between 4 and 5 PVP/GNPs, the absorbance values were largest. This indicates that graphene/water nanofluid with this mass ratio of PVP dispersant (i.e., 5 g PVP for every 1 g GNPs) could obtain better suspension stability. According to the optimum dispersant addition, nanofluid with four different GNPs concentrations of 0.01, 0.025, 0.05 and 0.075 wt % were prepared for further research. Figure 1c shows the prepared graphene/water nanofluid with different GNPs concentrations.

### 2.3. Thermophysical Properties

Plenty of literature has reported and suggested calculation correlations on the effects of adding nanoparticles on base fluid thermal conductivity and viscosity. In this study, thermal conductivity and viscosity of nanofluid with different concentrations were measured under different temperatures. A digital viscometer with measuring uncertainty of ±5% was used for measuring the viscosity of the nanofluid and deionized water at various given temperature conditions. The thermal conductivity measuring instrument works based on the principle of instantaneous linear heat source method and has an accuracy of ±5% in the temperature range from −50 °C to 150 °C. Temperature conditions of the tested fluid were controlled within ±0.1 °C temperature fluctuations, by using a thermostatic circulating water bath. 

**Figure 3:**
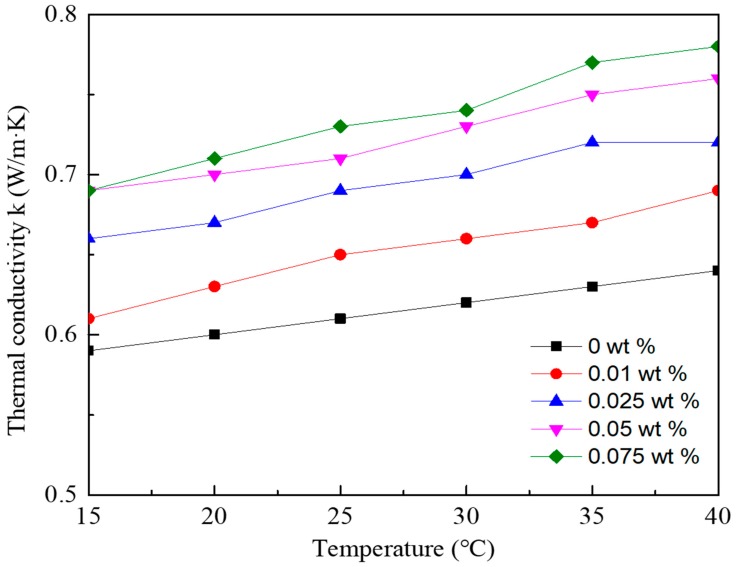
Thermal conductivity of graphene/water nanofluid with various GNPs concentrations at different temperatures.

Figure 3 shows that the thermal conductivities of both the graphene/water nanofluid and the deionized water increase with the increase of temperature. Meanwhile, the thermal conductivity of nanofluid increases as the GNPs concentration increases. For the nanofluid with a GNPs concentration of 0.075 wt %, the thermal conductivity reaches 0.71 W/(m·K), which is 18.6% higher than that of deionized water at the same temperature of 20 °C. 

Several theoretical models have been developed for predicting the thermal conductivity of nanofluid [3,4,28]. The theoretical calculations are relatively complicated, as many influence factors including the thermal conductivities of the base fluid and nanoparticles, volume fraction of particles, motion of dispersed particles and the matrix additive interface contact resistance should be taken into account. In this study, to evaluate the enhancement effects of adding nanoparticles, the thermal conductivity ratio of nanofluid to the base fluid is represented by a correlation equation as follows.

(2) $$\frac{k_{\mathrm{nf}}}{k_{\mathrm{bf}}}=(1+C_{k}w){\left(\frac{T_{\mathrm{nf}}}{T_{\infty }}\right)}^{C_{k,T}}$$

where *k*_nf_ and *k*_bf_ are the thermal conductivity of nanofluid and base fluid, respectively, in W/(m·K); *w* is the mass concentration of nanoparticles; *T*_nf_ is the temperature of nanofluid, in K; $T_{\infty }$ is the ambient temperature in K; *C*_k_ is the thermal conductivity enhancement coefficient; *C*_k,T_ is the thermal conductivity influence coefficient determined by temperature.

Using the measuring results, the relationship between the thermal conductivity ratio and GNPs concentrations at different temperatures was fitted and the coefficients in the equation can be concluded. Therefore, the correlation equation for the thermal conductivity ratio of graphene/water nanofluid is represented by Equation (3).

(3) $$\frac{k_{\mathrm{nf}}}{k_{\mathrm{bf}}}=(1+241w){\left(\frac{T_{\mathrm{nf}}}{T_{\infty }}\right)}^{1.0233}$$

The mean deviation between the calculated results of Equation (3) and measuring results is ±2.8%. 

**Figure 4:**
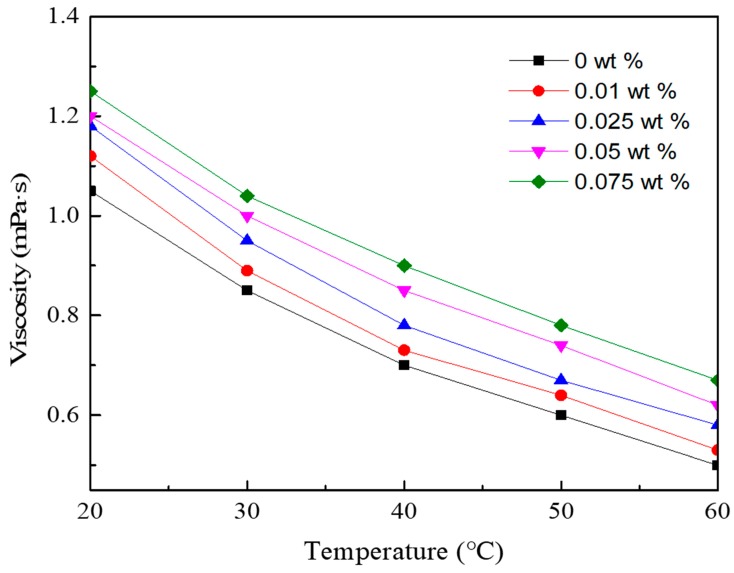
Viscosity of graphene/water nanofluid with various GNPs concentrations at different temperatures.

The viscosity of graphene/water nanofluid is shown in Figure 4. It is seen that the viscosity is closely related to the temperature. There is a remarkable decline in velocity when the temperature increases. In addition, it is clearly that loading GNPs would result in an increase of viscosity for the graphene/water nanofluid. Compared to the base fluid, the viscosity of nanofluid with GNPs concentration of 0.075 wt % was increased by 19% to 34% when the fluid temperature varies from 20 to 60 °C.

The relationship between the viscosity ratio and GNPs concentration of nanofluid at different temperatures was fitted and represented by correlation equation (Equation (4)). The mean deviation between the calculated results of Equation (4) and measuring results is ±8.0%.

(4) $$\frac{\mu_{\mathrm{nf}}}{\mu_{\mathrm{bf}}}=(1+373w-85566w^{2}){\left(\frac{T_{\mathrm{nf}}}{T_{\infty }}\right)}^{0.4573}$$

where *μ*_nf_ and *μ*_bf_ are the viscosity of nanofluid and base fluid, respectively, in mPa·s.

It is noted that the values of both the thermal conductivity and the viscosity of the base fluid at different temperatures can be calculated using the classic correlation equation for water [29], as the measured values for the base fluid (water) in this study agree well with the theoretical values.

In addition, it is observed that the enhancement ratio in viscosity for a graphene/water nanofluid with a specific GNPs concentration was nearly unchanged or increased slightly when the temperature increased. It agrees well with the finding of Reference [28] but has a little discrepancy with the result of Reference [29]. Reference [29] reported that the enhancement ratio in viscosity was slightly decreased with the increase of temperature. The discrepancy is caused by the difference in physical properties such as surface area of the GNPs. In addition, it is suggested that the values of parameters in Equations (3) and (4) are applicable for the experimented graphene/water nanofluid with a GNPs concentration ranged from 0.01 to 0.075 wt %, when the fluid temperature is between 15 to 60 °C.

The increase in the nanofluid’s thermal conductivity is beneficial for the enhancement of the phase-change heat transfer inside the heat pipe. However, the increase in viscosity is unfavorable. As for the nanofluid boiling heat transfer, it is affected by many factors such as the physical properties of nanofluid, roughness and hydrodynamics of the heated surface [18] and etc. Several works observed that the boiling heat transfer coefficient decreased as the concentration of nanoparticles increased. This is because the deposited nanoparticles sit on the heated surface and reduce the roughness and nucleation site density [32,33]. Taking into account that the stability of the suspension became worse with higher concentrations, a graphene/water nanofluid concentration range of not more than 0.05 wt % is preferred in solar gravity heat pipes for this study.

## 3. Results and Discussions

As it is operated in fluctuant solar radiation conditions, the solar gravity heat pipe may frequently start up and this severely affects its solar thermal collection performance. This study focused on the complicated start-up characteristics of a single solar gravity heat pipe, using graphene/water nanofluid as working fluid whose thermal properties has been measured in the previous section. In the first experiment, deionized water was used as the working fluid. This was done with the aim of obtaining the gravity heat pipe’s start-up characteristics and its general evaluation method. Based on this, the effects of using graphene/water nanofluid instead of deionized water on the solar gravity heat pipe’s thermal performance were experimentally investigated and comparatively discussed in the following section.

### 3.1. Description of the Solar Gravity Heat Pipes

Compared with an ordinary gravity heat pipe used for heat exchange, the gravity heat pipe used for solar collection exhibits a different geometric structure. Table 1 shows the design parameters of a solar gravity heat pipe used in experiments. The solar gravity heat pipe has a non-equal diameter, a long evaporation section with length of 1550 mm and inner diameter of 6 mm and a stubby condensing section with length of 50 mm and inner diameter of 12 mm for heat release. There is almost no adiabatic section because the transition from the evaporation to condensing section is not obvious. Both the evaporation and condensing section employ smooth cooper tubes without capillary wick inside. The condensing section is located above the evaporation section and the condensed liquid can drain back to the evaporation section by gravity instead of requiring a wick. Therefore, the solar gravity heat pipe transferring the absorber solar heat in one direction (from the bottom to the top) acts as a thermal diode to avoid reverse heat transfer loss. In the following experiments, the filling rate of working fluid was 18% of the total pipe volume for each heat pipe. 

### 3.2. Experimental Evaluation of the Start-Up Characteristics of a Single Solar Gravity Heat Pipe

#### 3.2.1. Experimental Setup

**Figure 5:**
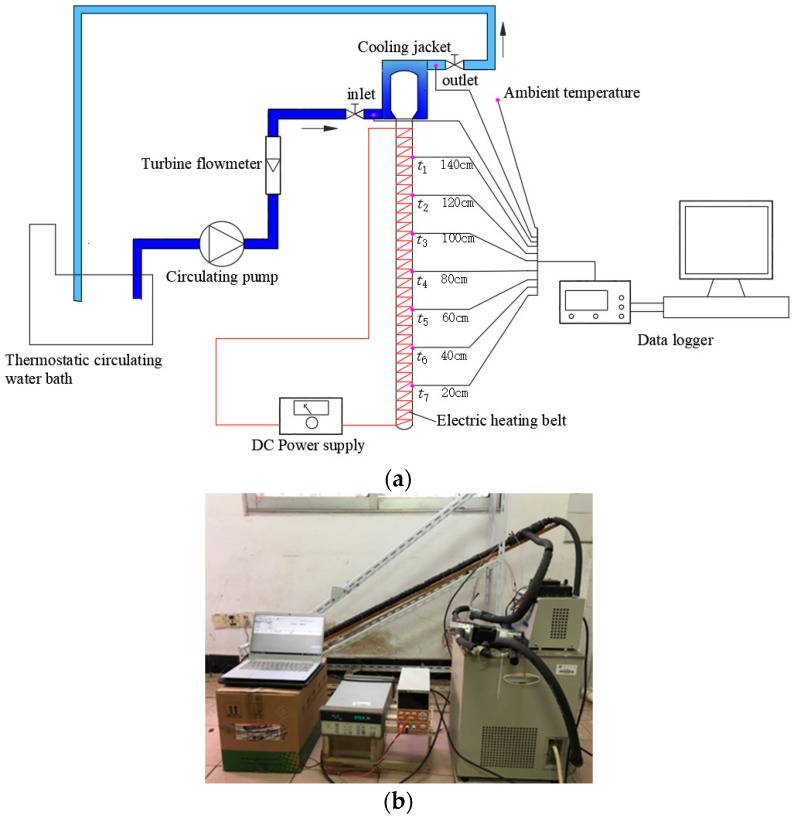
Experimental setup of the start-up characteristics testing for a single solar gravity heat pipe. (**a**) Schematic of the testing system; (**b**) A photograph of the experimental setup.

Figure 5 shows the schematic and experimental setup of the start-up characteristics testing for a single solar gravity heat pipe using deionized water as the working fluid. The experimental system mainly includes a single solar gravity heat pipe, thermostatic circulating water bath, electric heating belt and insulation material, direct-current power supply, circulating pump, plastic hose, data logger and computer. To simulate the actual working conditions for solar collection, the evaporation section of the heat pipe was heated using a tightly bound insulated electric heating belt. The operating characteristics of the heat pipe were investigated under different solar radiation levels by adjusting the heating power. Several T-type thermocouples with measuring accuracy of ±0.5 °C were adhered to the outer wall of the pipe for measuring the wall temperature at different height positions. The outside surface of the evaporation section was thermally insulated for preventing the heat loss to the ambient. The condensing heat was released to the water circulated from a thermostatic water bath to the cooling jacket of the condensing section. During the experiment, the water temperature was controlled at a given value with a fluctuation of less than ±0.5 °C. 

A common heat pipe solar collector operates under solar radiation conditions ranging from 200 to 1000 W/m^2^. The solar thermal heat flux absorbed by a single heat pipe is usually in the range of 10–70 W. In this experiment, the single solar gravity heat pipe was placed with an inclination angle of 30° and experimented under different heating power levels in the range of 20–70 W. This was done by adjusting the output voltage of the DC power supply. During the span of the experiment, the circulating water temperature was maintained at about 10 °C. The initial temperature of the heat pipe wall was basically the same as the circulating water temperature. 

#### 3.2.2. Principle of the Start-Up Process

**Figure 6:**
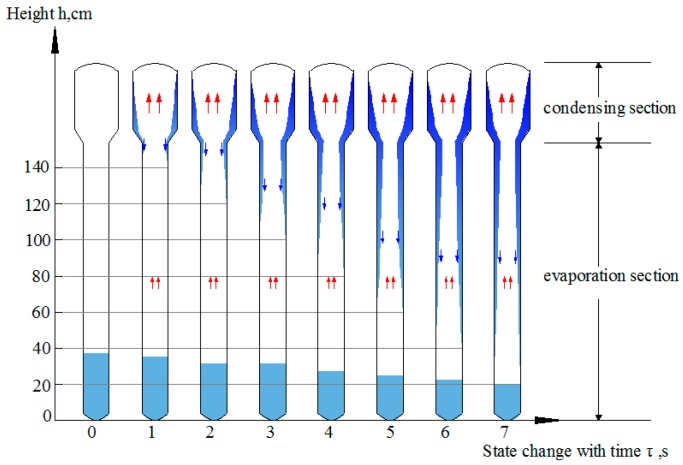
Diagram of the working state change during the start-up process of a solar gravity heat pipe.

After the power supply was turned on for a period of time, the heat pipe started up to work. The principle of the start-up process for the experimented solar gravity heat pipe is shown in Figure 6. It is outlined as follows. The initial liquid pool height inside the heat pipe in state 0 is approximately 37.15 cm as regard to a filling rate of 18%. At the beginning of heating, there is little condensate due to the little vaporized steam and this floats upwards. With continuous heating in the evaporation section, the amount of vaporized steam inside the heat pipe increases and releases heat to the water in the condensing section. Consequently, the amount of condensed reflux liquid increases. When the reflux liquid flows to a height of 140 cm, as shown in state 1, the wall temperature at this height suddenly drops to the level of the condensing temperature. Similar to what is described above, the condensate reflux liquid gradually flows through the heights of 120, 100, 80, 60 and 40 cm and the resultant temperatures sequentially drop to the levels of the corresponding condensing temperatures as heating goes on. When it finally flows to the height of 20 cm, the amount of vaporized steam and condensate reaches a steady state 7 without any change. The heat pipe thus completes the start-up process. 

#### 3.2.3. Thermal Start-Up Characteristics of a Single Gravity Heat Pipe 

**Figure 7:**
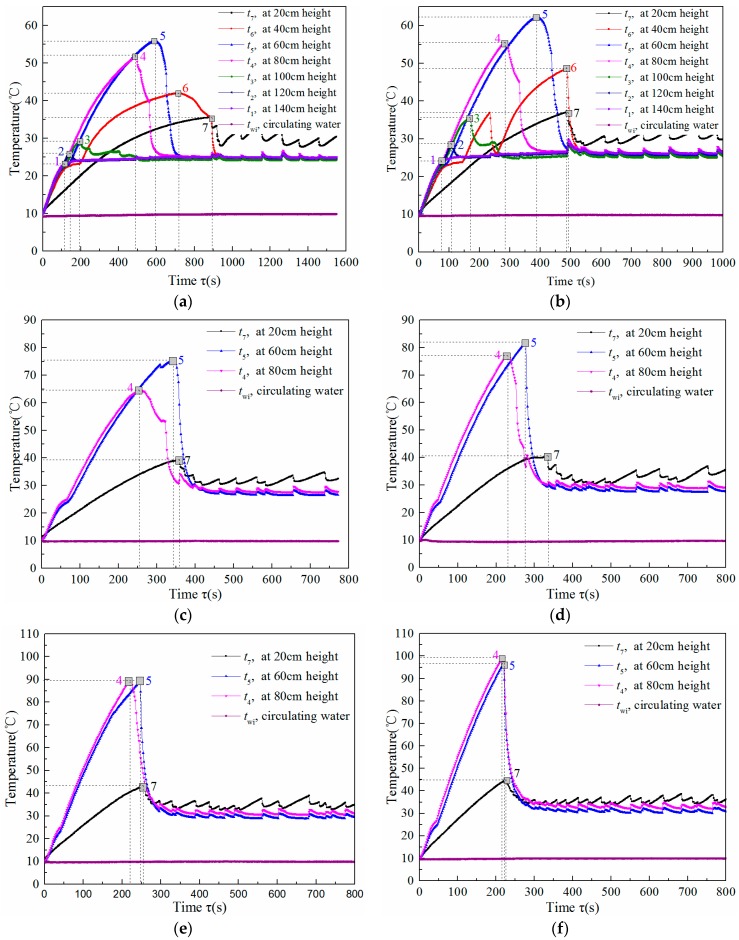
Variations of pipe wall temperatures throughout operation time under different heating power conditions: a heating power of (**a**) 20; (**b**) 30; (**c**) 40; (**d**) 50; (**e**) 60 and (**f**) 70 W.

The variations of pipe wall temperatures at different heights after different heating time during the start-up period was experimentally investigated and the results are shown in Figure 7. This verified the aforementioned start-up process of the heat pipe. Figure 7a shows the variations of pipe wall temperature as the heat pipe operates with a heating power of 20 W. When the heating is started, the wall temperatures at the different heights increase rapidly. This is because most of the input heat is used to heat the pipe wall and the initial liquid pool height inside the heat pipe. As the heating continues, vaporized steam increases and floats up to the condensing section. Then, the condensate flows back down. When the condensed reflux liquid flows to the height of 140 cm, the temperature at this height (*t*_1_) encounters the inflection point 1 shown in the figure, sharply drops in temperature to match the condensate temperature and then remains at that temperature. After this, the wall temperatures at the evaporation section from top to bottom reflect their corresponding inflection points in the order of point 2, 3, 4, 5, 6 and 7. Before the time point 7 is reached, the heat pipe completes the start-up and later works in a steady state. As the height of 20 cm is near the liquid pool inside the heat pipe, the wall temperature at this height (*t*_7_) has certain fluctuations. Figure 7b–f shows similar variation tendencies of pipe wall temperatures when the heat pipe was operated under different heating power conditions. Only parts of the heat pipe wall temperature curves were illustrated in the figures for brevity. 

Based on the principle presented in Section 3.2.2, the heat pipe’s start-up time (∆*τ*_s_) was defined as the time when inflection point 7 appeared. When the heating power was 20, 30, 40, 50, 60 and 70 W, the starting time of the heat pipe was 890, 490, 356, 334, 248 and 226 s, respectively. The start-up time was shortened with the increase of heating power. That is, a lower solar radiation operation requires a longer time to start up. In addition, there is a sudden increase in the wall temperature during the start-up period. The maximum wall temperature (*t*_s_) corresponding to the given heating power levels was 55.7, 62.0, 75.5, 81.9, 89.0 and 97.1 °C, respectively. 

### 3.3. Thermal Start-Up Performance of Nanofluid-Enhanced Solar Gravity Heat Pipe for Water Heating

#### 3.3.1. Comparative Experiment 

Using the start-up characteristics evaluating method and the defined start-up time mentioned in the above section, comparative experiments between the thermal performance of the nanofluid enhanced solar gravity heat pipes and that of the conventional deionized water solar gravity heat pipes were carried out. 

**Figure 8:**
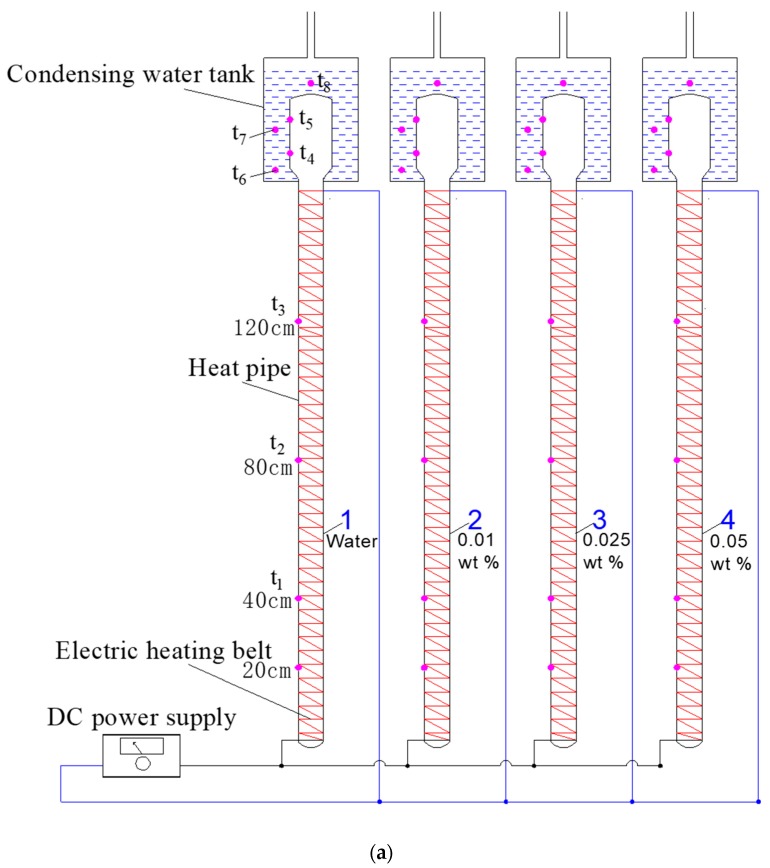

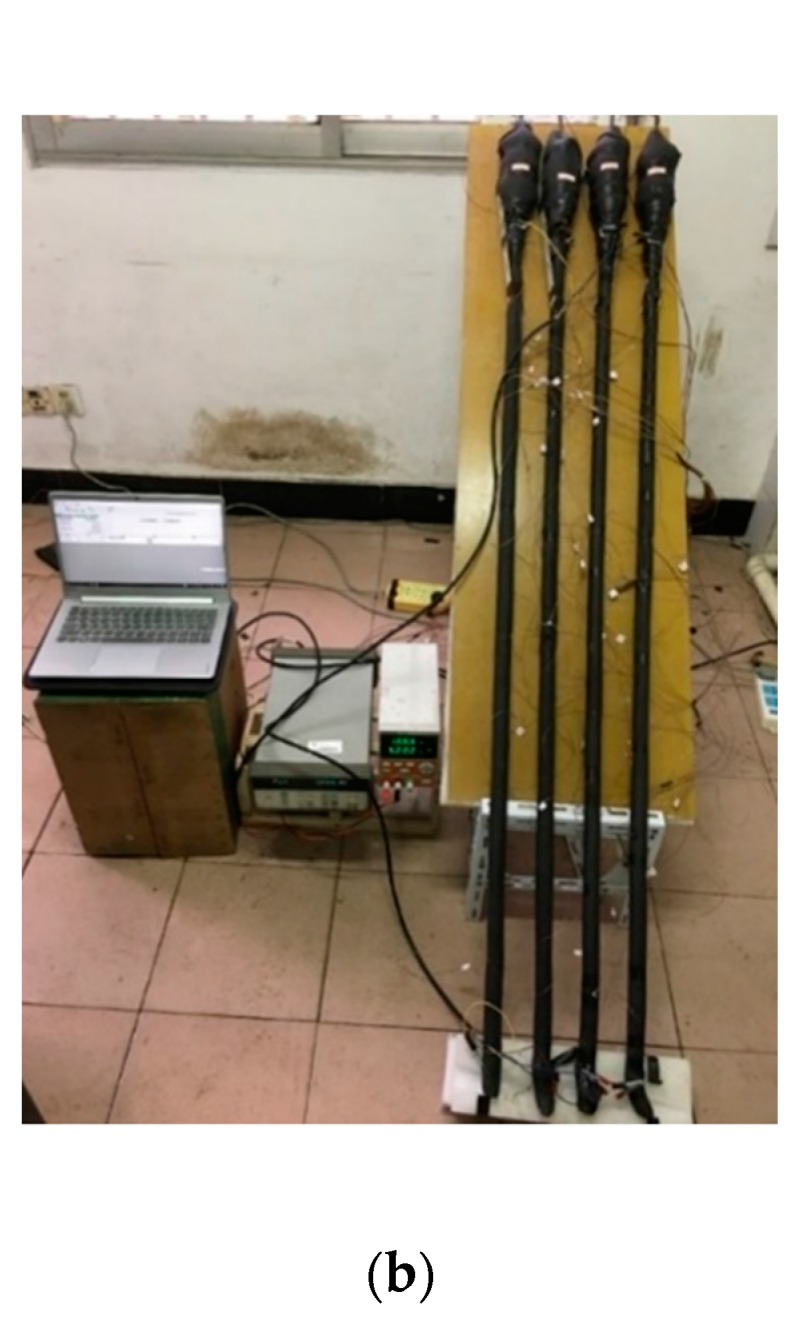
Schematic diagram and setup of the comparative experiment for solar gravity heat pipes. (**a**) Schematic diagram; (**b**) Photograph of the experimental setup.

The schematic diagram and setup of the comparative experiment are shown in Figure 8. Three heat pipes filled with graphene/water nanofluid with 0.01, 0.025 and 0.05 wt % GNPs concentrations and one heat pipe filled with deionized water were placed at the same inclination angle of 30°. The filling rates of the working fluid for the four heat pipes were the same. Also, the heating power input (*E*) to the heat pipes’ evaporation sections were the same and controlled by adjusting the output voltage of the DC power supply. The layout of thermocouples for measuring pipe wall temperatures at different heights of 40, 80 and 120 cm (namely *t*_1_, *t*_2_ and *t*_3_, respectively) are shown in the figure. In obtaining the heat pipe’s start-up time, it is important to note that the wall temperature at 20 cm height has certain fluctuations. However, the time when the wall temperature inflection point at 40 cm height appears is very close to that at the height of 20 cm. Therefore, the time when the wall temperature inflection point appeared at 40 cm height was regarded as the start-up time (*τ*_s_).

To evaluate the heat transfer capacity of the heat pipes, the released condensing heat of working fluid was used to heat water. Since the heat transfer capacity of a single solar gravity heat pipe was very low, the condensing sections of each heat pipe were immersed in a mini condensing water tank filled with a same volume of water. Thermal insulation layer was adhered at the outside of the water tank. Ignoring the heat loss to ambient, the temperature rise of the water measured within a set operation time duration (∆*τ*) was used to calculate the heat transfer capacity as shown in Equation (5). The water temperature (*t*_w_) was calculated as the average temperature at three measure points located in the mini condensing water tank. The accuracy of the thermocouples used for measuring water temperature was ±0.3 °C.

(5) $$Q_{w}=\rho VC\Delta t_{w}$$

where *Q*_w_ is the heat gain of water measured in Joules, *ρ* is the density of the water in m^3^/kg, *C* is the specific heat capacity of the water in m^3^/kg, *V* is the volume of the mini condensing water tank in m^3^ and ∆*t*_w_ is the temperature rise of the water in °C.

The thermal efficiency of the single heat pump was calculated using

(6) $$\eta =\frac{Q_{w}}{E\Delta \tau }$$

where *E* is the heating power input to the heat pipe in Watts and ∆*τ* is the operation time duration in seconds.

#### 3.3.2. The Effect of Start-Up Time on the Thermal Efficiency of the Heat Pipe for Heating Water

**Figure 9:**
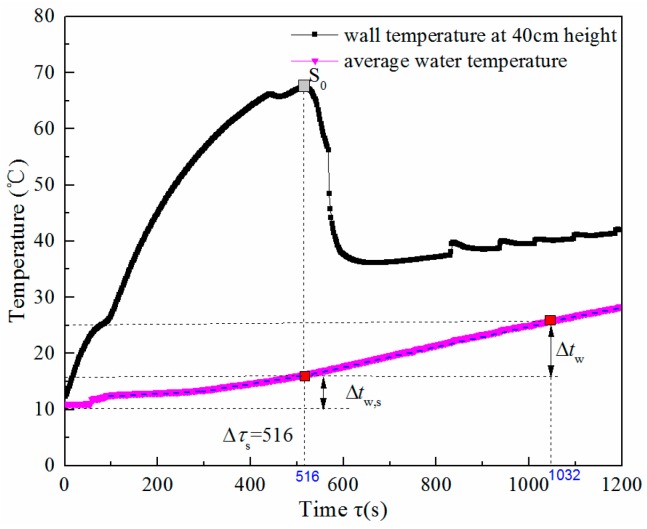
Variation of water temperature over time for the deionized water heat pipe.

Figure 9 shows the average water temperature rise in the mini condensing water tank when the deionized water heat pipe operates under 30 W heating power. It is seen that *t*_w_ rises continuously over time. The heat pipe is in the thermal start-up process during the time 0 *≤* *τ* *≤ τ*_s_. Following this, the heat pipe operates in a steady stage. The experimental results indicate that the water temperature rise in the start-up period (∆*t*_w,s_) was 5.2 °C, while ∆*t*_w_ in the steady state period was 11.0 °C after operating during the same time interval (*τ*_s_ *≤* *τ* *≤* 2*τ*_s_). It demonstrated that the thermal efficiency of the heat pipe operating in the steady period (*η*) was higher than that in the start-up period (*η*_s_). According to Equations (5) and (6), *η*_s_/*η* is equal to the ratio between the *∆t*_w,s_ and *∆t*_w_. This is valued at 0.47 in the above operating conditions.

To analyze the effect of the start-up time on the thermal efficiency of the heat pipe given a set operation time (duration ∆*τ*) when the heat pipe only goes through one start-up process, the average thermal efficiency during the time interval can be evaluated using

(7) $$\bar{\eta }=\frac{Q_{w,s}+Q_{w}}{E\Delta \tau }=\frac{\Delta \tau_{s}\eta_{s}+(\Delta \tau -\Delta \tau_{s})\eta }{\Delta \tau }=\eta \left[1-\frac{\Delta \tau_{s}}{\Delta \tau }(1-\frac{\eta_{s}}{\eta })\right]$$

where *Q*_w_ and *Q*_w,__s_ are the heat gain of water (in Joules) when the heat pipe is operated during the steady period and during the start-up period, respectively; *η* and *η*_st_ are the thermal efficiency of the heat pipe operating during the steady period and the start-up period, respectively; ∆*τ*_s_ is the heat pipe’s start-up time in seconds; and ∆*τ* is the operating time duration in seconds when the heat pipe only goes through one start-up process.

Based on the above experimental condition,

(8) $$\bar{\eta }=\eta \left[1-0.53\frac{\Delta \tau_{s}}{\Delta \tau }\right]$$

Therefore, in a given operation time duration, the shorter the start-up time is, the higher the average thermal efficiency is. 

#### 3.3.3. Thermal Start-Up Performance of the Solar Heat Pipe Using Nanofluid 

The start-up characteristics for solar heat pipes using nanofluid and deionized water were compared experimentally. During the experiments, the heating power was controlled at 30 and 60 W, respectively. The initial water temperature in the mini condensing water tank was 11 °C and the ambient temperature was maintained at about 12 °C.

**Figure 10:**
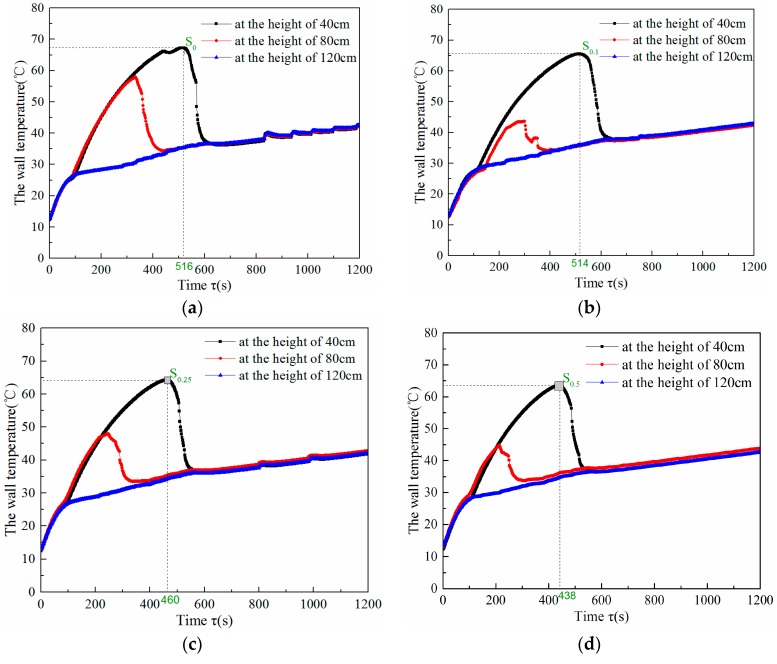
Variations of wall temperatures throughout the duration of the experiment for heat pipes filled with different kinds working fluid under 30 W heating power. (**a**) Deionized water heat pipe; (**b**) Nanofluid enhanced heat pipe with a 0.01 wt % concentration of GNPs; (**c**) Nanofluid enhanced heat pipe with a 0.025 wt % concentration of GNPs; (**d**) Nanofluid enhanced heat pipe with a 0.05 wt % concentration of GNPs.

Figure 10 shows the variations of wall temperatures over time for heat pipes filled with different working fluids under 30 W heating power. As seen in Figure 10a, the *∆τ*_s_ for the deionized water heat pipe was 516 s and the corresponding maximum wall temperature occurred in the start-up process (*t*_s_) was 67.3 °C. In contrast, when using graphene/water nanofluid with GNPs concentrations of 0.01, 0.025 and 0.05 wt %, the *∆τ*_s_ for nanofluid-enhanced heat pipes was shortened to 514, 460 and 438 s, respectively. The corresponding *t*_s_ decreased to 65.5, 64.3 and 63.6 s for those concentrations, respectively. This is mainly attributed to the factors listed as follows. (1) The addition of GNPs increases the thermal conductivity of the base fluid which has been confirmed by the measure results presented in Section 2.3 and reduces the surface tension of working fluid [29]. (2) The Brownian motion of graphene nanoplatelets, the deposition of nanoplatelets on the smooth surface of the evaporation pipe increase the number of small vapor bubbles instead of a few large bubbles [18]. These help to enhance the nucleate pool boiling inside the heat pipe. As a result, the vapor generation became faster and thus the start-up process was accelerated. 

Additionally, the start-up time (*∆τ*_s_) and corresponding maximum wall temperature (*t*_s_) for the solar heat pipes operating under 30 and 60 W of heating power are listed in Table 2. When using 0.05 wt % graphene/water nanofluid instead of deionized water, a 15.1% and 10.7% reduction in start-up time was achieved under 30 and 60 W heating conditions, respectively. Meanwhile, the maximum wall temperature occurred in the start-up process was decreased by 3.7 and 7.7 °C, respectively, suggesting that lower absorber temperatures were required for the start-up of gravity solar heat pipes. It is observed that the improvement of the start-up performance is more effective when the nanofluid-enhanced heat pipe operates under a lower heating power condition. In addition, within the concentration range of 0.01–0.05 wt %, a higher GNPs concentration for the nanofluid obtains a better enhancement effect.

According to the analysis in Section 3.2.2, shortening the start time of the heat pipe can improve its thermal efficiency. Since solar radiation exhibits a large fluctuation and a low heat flux, especially on cloudy days, a heat pipe solar collector operating outdoors probably undergoes intermittent shutdowns and restarts. Therefore, the use of nanofluid as the working fluid instead of deionized water could significantly shorten the overall start-up time of gravity heat pipes and be helpful to start the heat pipes under low heating conditions and, consequently, create a higher thermal efficiency for solar collection. 

## 4. Conclusions

To enhance the thermal performance of a solar gravity heat pipe, graphene/water nanofluid with different concentrations were prepared and used as the working fluid instead of deionized water. The thermal start-up performance of nanofluid-enhanced solar gravity heat pipes were experimentally investigated and compared with a conventional solar heat pipe. The main findings of this study are as follows

- An optimal dispersant addition for graphene/water preparation was obtained. The mass ratio of 5 g PVP to 1 g GNPs exhibits the best dispersion stability. The addition of GNPs to deionized water increases both thermal conductivity and viscosity. Correlations have been given to evaluate the effects of GNPs concentration on the thermal conductivity and the viscosity of graphene/water nanofluid at temperatures ranged from 20 to 60 °C.
- In the range of 0.01–0.05 wt %, the higher the GNPs concentration, the greater the enhancement effect on the thermal start-up process. When 0.05 wt % grapheme/water nanofluid was used as working fluid instead of deionized water, the start-up time of a solar gravity heat pipe could be shortened by 15.1% and 10.7% under 30 and 60 W input heating conditions, respectively.
- In the solar heat pipe for water heating, the thermal efficiency during the start-up period was much lower than that in the steady operation period. Consequently, a higher thermal efficiency for solar collection could be expected when the start-up time was significantly reduced.

Results in this study demonstrate the superiority of using nanofluid to enhance the thermal start-up performance of a solar gravity heat pipe. Further investigations should be carried out on the overall efficiency of the nanofluid-enhanced heat pipe solar collector.

## Figures and Tables

**Table 1:** The design parameters of a solar gravity heat pipe used in experiments.

Components	Materials	Length (mm)	Inner Diameter (mm)	Outer Diameter (mm)
single heat pipe	copper	1600	—	—
evaporation section	copper	1550	6	8
condensing section	copper	50	12	14

**Table 2:** The start-up time for solar heat pipes under heating powers of 30 and 60 W.

Working Fluids	GNPs Concentrations	*∆τ*_s_ (s)		*t*_s_ (°C)	
		30 W Heating	60 W Heating	30 W Heating	60 W Heating
deionized water	0	516	330	67.3	84.2
nanofluid	0.01 wt %	512	312	65.5	82.0
nanofluid	0.025 wt %	460	304	64.3	80.7
nanofluid	0.05 wt %	438	298	63.6	76.5
